# Social Norms Shift Preferences for Healthy and Unhealthy Foods

**DOI:** 10.1371/journal.pone.0166286

**Published:** 2016-11-18

**Authors:** Emma M. Templeton, Michael V. Stanton, Jamil Zaki

**Affiliations:** 1 Department of Psychological and Brain Sciences, Dartmouth, Hanover, New Hampshire, United States of America; 2 Health Sciences Program, California State University, East Bay, Hayward, California, United States of America; 3 Department of Psychology, Stanford University, Stanford, California, United States of America; TNO, NETHERLANDS

## Abstract

This research investigated whether people change their food preferences and eating behavior in response to health-based social norms. One hundred twenty participants rated a series of healthy and unhealthy food images. After each rating, participants sometimes viewed a rating that ostensibly represented the average rating of previous participants. In fact, these average ratings were manipulated to convey a particular social norm. Participants either saw average ratings that favored healthy foods, favored unhealthy foods, or did not see any average ratings. Participants then re-rated those same food images after approximately ten minutes and again three days later. After the norm manipulation, participants were given the chance to take as many M&Ms as they wanted. Participants exposed to a healthy social norm consistently reported lower preferences for unhealthy foods as compared to participants in the other two conditions. This preference difference persisted three days after the social norm manipulation. However, health-based social norm manipulations did not influence the amount of M&Ms participants took. Although health-based social norm manipulations can influence stated food preferences, in this case they did not influence subsequent eating behavior.

## Introduction

People who primarily consume whole grains, fruits, and vegetables typically live healthier, longer lives than people who primarily consume saturated fat and added sugars [1]. Increased availability and consumption of unhealthy foods is one factor contributing to an increase in obesity [2], a disease linked to many serious health conditions and mortality [3]. As such, a better understanding of factors influencing eating behavior can pave the way for targeted interventions that can encourage healthier eating and ultimately improve quality of life.

Here, we focus on social factors that influence food consumption, particularly *social norms*. Social norms are the rules that define the values, beliefs, and behaviors of a given group [4]. Scientists typically describe two types of norms: injunctive and descriptive. *Injunctive* norms describe one’s perception of what other people think we *should* do in a given situation whereas *descriptive* norms describe one’s perception of what most people *actually* do in a given situation. When it comes to food consumption, the injunctive norm is likely health-positive (i.e., People should eat healthy foods and limit their consumption of unhealthy foods). However, the descriptive norm is less straightforward.

Descriptive norms can powerfully influence behavior, even when people fail to see their importance [5]. Consider energy conservation. When asked to rank a list of reasons for conserving energy at home, residential energy users rated environmental, societal, and financial benefits above social norms. In actuality, people who believed that others were conserving energy engaged in more energy saving efforts themselves [6,7]. Descriptive norms predicted energy conservation more than any other motivator. Manipulating perceptions of social norms can have important, real-world consequences in many domains. For instance, telling people that their friends are voting in an election can encourage more people to vote [8,9], and telling college students that their peers avoid binge drinking has the ability to reduce problematic drinking behavior, potentially saving lives [10,11].

People overwhelmingly believe that their eating behavior reflects non-social factors including their hunger or satiety and their idiosyncratic taste preferences [12,13]. However, social norms often exert powerful effects on individuals’ eating [14]. Studies that manipulate perception of these norms typically employ a *remote confederate* paradigm [15–18]. In this paradigm, participants are led to believe that previous participants ate either a large amount or a small amount of a particular food item—cookies, doughnuts, pizza, etc. The participant is then permitted to eat as much or as little of that particular food item as they want. These studies consistently find that participants consume amounts similar to the remote confederates before them, suggesting that remote confederate behavior influenced the participants’ behavior.

Remote confederate studies provide important demonstrations of descriptive norms influencing eating behavior, but the inferences scientists can draw from these paradigms are limited. In these studies, participants are exposed to a social norm about one specific food item, as opposed to general “rules” governing their peers’ eating behavior. As such, it remains unclear whether participants who learn that remote confederates ate a small amount of one food (e.g., cookies) would generalize this knowledge to a broader social norm and also eat less of a second unhealthy food (e.g., pizza). Further, these studies investigate only the immediate effects of social norm manipulations, and thus do not clarify the extent to which norm-based food preferences persist over time. One obvious direction for this work is the construction of norm-based interventions to encourage healthy eating. Intervention studies that have investigated whether descriptive norms can be used to encourage healthier eating have had mixed results [19]. In order to assess the effectiveness of such interventions, it is important to first assess whether the effects of social norm manipulations can be generalized to related stimuli and whether they persist over time.

The present study addressed these limitations by adopting a paradigm we and others have used to study social influence [20–25], including over food preferences [22]. In this recent study, participants rated their preferences for healthy and unhealthy food images and then saw the average rating that previous participants ostensibly gave those same food images. For example, a participant might rate a picture of spinach as “7” and then see that the average rating for spinach was a “4.” These average ratings were manipulated such that roughly 1/3 of responses were higher than the participant’s initial rating, 1/3 were lower than the participant’s initial rating, and 1/3 matched the participant’s initial rating. Participants then rated the same set of food images a second time, without viewing average ratings. During these second ratings, participants changed their food preferences to align with those of the group [22]. Crucially, though each participant rated both healthy and unhealthy images, peer ratings varied independently of the healthfulness of the stimuli. A participant might learn that their peer group liked chocolate cake and spinach equally. Interestingly, participants exhibited equal levels of susceptibility to social influence over both healthy and unhealthy foods.

The present study modified this paradigm to investigate whether social norms not only alter preferences for *specific* food items, but can also change participants’ preferences for an entire *category* of foods: healthy versus unhealthy. To do this, we manipulated average peer ratings so that they followed a clear rule. Participants were randomly assigned to one of three conditions and either observed peer ratings that favored healthy foods (Healthy Norm condition), observed peer ratings that favored unhealthy foods (Unhealthy Norm condition), or saw no peer ratings (No Norm condition). Participants then completed two follow-up rating sessions. Unlike previous conformity paradigms, these re-rating sessions included novel healthy and unhealthy food items that participants did not see in the first rating session. The second re-rating session took place three days after the norm manipulation.

This modified paradigm addressed the limitations in remote confederate studies by focusing on the generalizability and persistence of social norms. In this design, participants had to learn the norm rule by integrating norm information for a range of food items. Participants then had to apply this rule to novel stimuli. Comparing follow-up ratings for food items in the first task that were associated with norm information (repeated images) with ratings for images not in the first task and therefore not associated with norm information (novel images) allowed us to better understand how people encode rule-based social norm information. If participants only updated their preferences on an item-by-item basis, ratings for repeated images should align with group norms, but ratings for novel images should not. However, if participants learned and generalized a norm rule (e.g., the group prefers healthy foods), ratings for repeated *and* novel images should both align with group norms. Further, the second follow-up session made it possible to determine whether the effects of the norm manipulation persisted three days later. We predicted that participants would conform to the rule-based norm manipulation by aligning their preferences to the group ratings, that these preference differences would generalize to novel stimuli, and that this conformity would persist three days later.

Finally, we investigated potential additional consequences of this novel social norm manipulation. Although plenty of evidence suggests that conformity paradigms like the one we employ here cause participants to change their stated preferences, it is not known whether this translates into any other related changes. Changing stated food preferences in a computer task does not necessarily mean that participants will similarly change their eating behavior. Thus, we investigated changes in eating behavior by giving participants the opportunity to take as much of an unhealthy, palatable food as they wanted before leaving the testing session. We also investigated changes in health perception by asking participants to rate how healthy they believed each food item to be. Health perception can influence eating behavior [26–28] and might be more susceptible to social influence. Investigating health perceptions and real-world eating behavior helped us understand what potential this social norm manipulation might have as an intervention strategy to encourage healthier eating. More generally, investigating how participants respond to health norms can teach us more about how descriptive norms are transmitted from a group to an individual.

## Method

### Participants

Participants signed up to attend two testing sessions, in exchange for monetary compensation. One hundred twenty (84 female) non-dieting, non-vegetarian Stanford students between 18 and 25 years old participated in the first testing session. Participants were an average age of 19.9 years old (*SD* = 1.67) and had an average BMI of 22.0 (*SD* = 2.44). Participants in the Healthy Norm condition (29 female) were an average age of 20.08 years old (*SD* = 1.53) and had an average BMI of 21.15 (*SD* = 2.15). Participants in the Unhealthy Norm condition (24 female) were an average age of 19.83 years old (*SD* = 1.81) and had an average BMI of 22.51 (*SD* = 2.46). Participants in the No Norm condition (31 female) were an average of 19.78 years old (*SD* = 1.69) and had an average BMI of 22.41 (*SD* = 2.50). There were no differences in age or gender between conditions. However, participants in the Healthy Norm condition had significantly lower BMI than participants in the Unhealthy Norm (*p* = .01) and No Norm (*p* = .02) conditions. Because participants were assigned conditions randomly, this represents a failure of randomization. BMI was not a significant predictor of any outcome variables and was therefore not included as a covariate. Further, BMI did not moderate the effect of condition on any outcome variables. Two participants failed to attend the second testing session and one participant was unable to complete the final task due to a computer failure. Stanford’s Institutional Review Board approved the study and all participants provided informed, written consent.

### Procedure

Participants completed four image-rating tasks—two during the first testing session, and two during a second testing session three days later.

#### Testing Session #1

**Fig 1:**
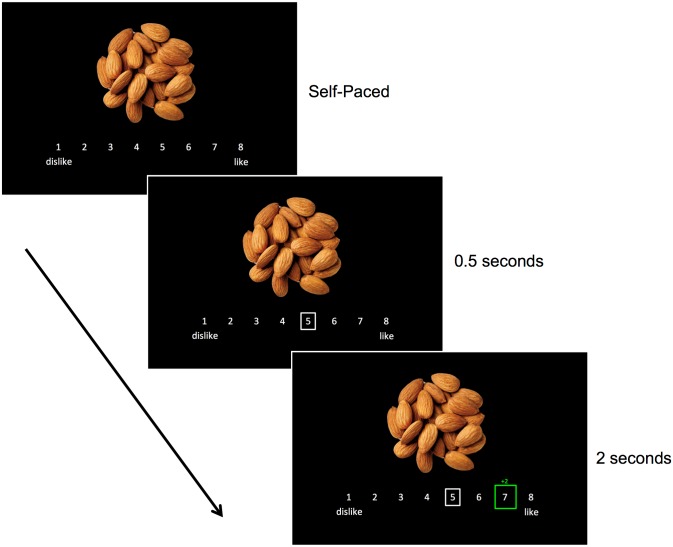
Task design. During each trial, participants viewed and rated their preference for a food item. Participants could take as long as they liked to rate their preferences, though each food image remained on the screen for at least 2 seconds. If participants took longer than 2 seconds to respond, the image remained on screen for an additional 0.5 seconds. Afterward, the peer rating appeared for 2 seconds. The difference between the participant’s rating and the peer rating was always indicated above the green box. When the ratings were identical the text said, “agree.”

In the first task, participants rated their preferences for a series of 180 food images. Half of these images depicted healthy foods (e.g., grapes, green beans, etc.; average of 132 calories per 100 grams) and the other half depicted unhealthy foods (e.g., chips, cookies, etc.; average of 352 calories per 100 grams). The nutritional qualities of foods in these groups differed significantly (all *ps* < .001). Participants made self paced ratings of their preferences for each food on an 8-point Likert scale (1 = dislike, 8 = like). After making each rating, participants viewed the average rating for that same food item, ostensibly calculated from ratings of 200 other Stanford students. We will refer to this as the *peer rating*. The participant’s rating and the peer rating appeared on the same scale for two seconds, highlighting any differences between the two ratings (Fig 1).

Peer ratings were manipulated to adhere to one of two health norms. Participants in the Healthy Norm condition (N = 40) saw peer ratings that favored healthy foods, whereas participants in the Unhealthy Norm condition (N = 40) saw peer ratings that favored unhealthy foods. *Favored* foods (i.e., healthy foods in the Healthy Norm condition and unhealthy foods in the Unhealthy norm condition) were paired with peer ratings that had a mean of 6.5 (moderate-high liking on the 8 point scale) and a standard deviation of 1, whereas *un-favored* foods (e.g., unhealthy foods in the Healthy Norm condition) were paired with peer ratings with a mean of 2.5 and a standard deviation of 1. To increase credibility, peer ratings were never ‘1’ or ‘8,’ as it would be unlikely for an “average” preference to be on the either extreme end of our scale. These extreme ratings have damaged believability in prior work (Zaki, Schirmer, & Mitchell, 2011). Participants in the No Norm condition (N = 40) did not see any peer ratings, and acted as a control group.

After a 10-minute filler task of responding to survey items, participants completed the second image-rating task. Here, participants rated their preferences for 180 food images. Again, 90 of these food images depicted healthy foods and 90 of these food images depicted unhealthy foods. 120 of these images were repeated items from the first rating task and therefore had been previously paired with peer ratings. The remaining 60 images were novel images (30 healthy foods and 30 unhealthy foods) that had not been previously paired with peer ratings.

After the re-rating task, participants were excused from the first testing session. On their way out, participants were encouraged to take M&Ms from a jar. The jar could hold 1.5 quarts (1420 ml) and was nearly filled with M&Ms. After the experimenter offered M&Ms to each participant she intentionally turned around to work on a computer behind a divider, obscuring her view of the participant. This created the illusion that the experimenter would not know how much candy participants took, thus encouraging participants to eat as much as they liked [18]. In fact, we weighed this jar before participants arrived and after they left to determine the amount of M&Ms each participant took. This is a widely-used measure of eating behavior [29–35]. Though this measure is not a comprehensive assessment of eating behavior, it serves as a simple assessment of whether or not social norm information about health preferences might impact eating behavior. Because liking a food is a good predictor of eating it [36], we predicted that participants who show an increased preference for unhealthy foods would consume more M&Ms.

#### Testing Session #2

Three days after completing the first testing session, participants returned to the lab for their second testing session. First, participants rated their preferences for all 240 food images (180 images from the first rating task and the 60 novel images from the second rating task).

Finally, participants completed the fourth image-rating task, where they rated all 240 images once again. Instead of rating their preference for each food, participants rated how *healthy* they believed each food to be, using an 8-point Likert scale (1 = unhealthy, 8 = healthy). This was our measure of health perception.

## Results

### Conformity

First we determined whether participants *conformed*: that is, adjusted their ratings to align with peer ratings on an item-by-item basis. To investigate this, we applied an analysis used in our prior work [22], as well as other recent conformity research [24]. We grouped each trial into one of three feedback bins: Peers Higher (25.8% of all trials), Peers Lower (31.4% of all trials), and Peers Same (42.8% of all trials). These conditions included, respectively, trials on which peer ratings were 2 or more points higher, 2 or more points lower, or within one point of the participant’s own initial rating. We then computed the amount that participants shifted their rating of items in each bin between initial and follow-up ratings. For instance, if participants increased their rating of a food in the *peers higher* condition (which were associated with high group ratings), this would indicate conformity to the group. We conducted a mixed effects analysis to compare participants’ rating shift across trial type, entering feedback type (i.e., Peers Lower, Peers Same, Peers Higher) as a fixed effect and participant as a random effect. We also entered participants’ initial ratings for each trial as a fixed effect covariate to control for the possibility that our results could be explained by regression to the mean [37]. Note that this analysis did not include information about the type of food (healthy versus unhealthy) participants viewed in each trial.

**Fig 2:**
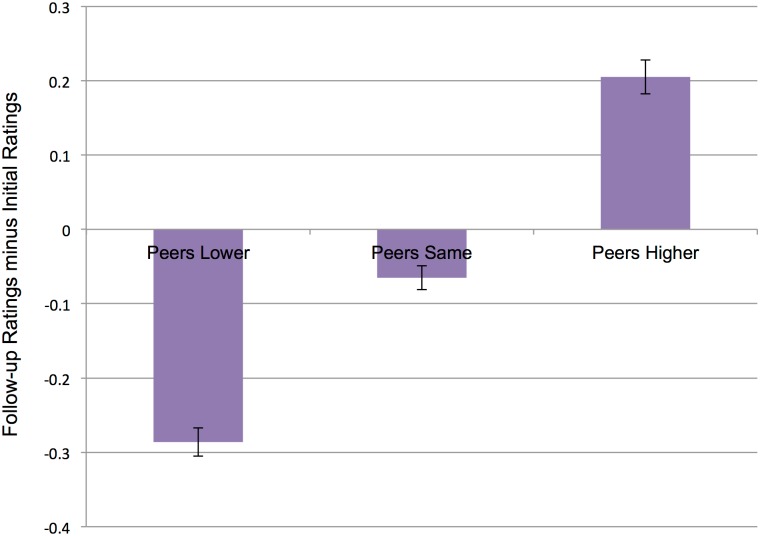
Participants shift preference ratings to conform to peer ratings. Participants decreased their preferences for items that their peers rated lower (Peers Lower) and the same (Peers Same). Participants increased their preferences for items that their peers rated higher (Peers Higher). This figure includes all trials from all participants. Error bars represent *SEM*.

Consistent with a conformity account, participants’ follow-up ratings shifted to align with peer ratings. Participants decreased their ratings for foods that their peers rated lower (*M* = -0.29, *t* = -3.23, *p* < .01) and increased their ratings for foods that their peers rated higher (*M* = 0.21, t = 2.88, *p* < .01; Fig 2). Participants also decreased their ratings for foods that their peers rated the same (*M* = -0.07, t = -2.93, *p* < .01), though this decrease was significantly less than the decrease for foods that their peers rated lower (*t*(79) = 6.19, *p* < .001, *d* = .70).

### Preferences

**Fig 3:**
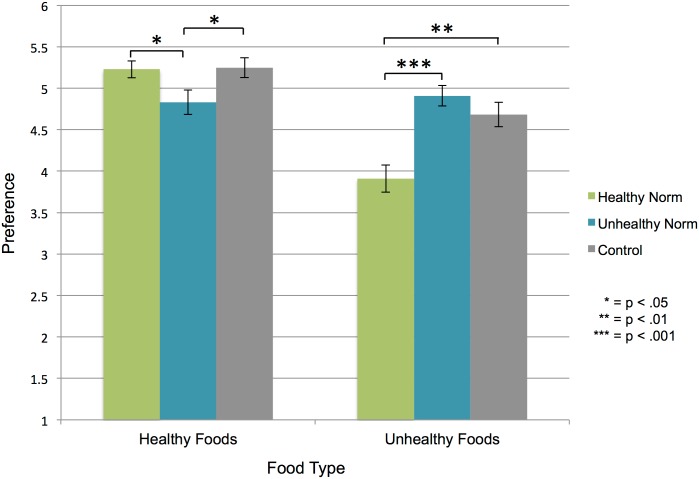
Initial preference ratings align with manipulated norms. Preference ratings during the norm manipulation show that participants in the Healthy Norm condition have a greater preference for healthy foods than participants in the Unhealthy Norm condition and that participants in the Unhealthy Norm condition have a greater preference for unhealthy foods than participants in the Healthy Norm condition. Error bars represent *SEM*.

After establishing that participants shifted their preferences in response to social norms for individual items, we then investigated whether participants shifted their preferences for entire *categories* of foods. We expected that participants in the Healthy Norm condition would show an increased preference for healthy foods and participants in the Unhealthy Norm condition would show an increased preference for unhealthy foods. As predicted, there was a statistically significant difference between conditions for preferences for healthy foods (*F*(2,117) = 3.61, *p* = .03) as well as preferences for unhealthy foods (*F*(2,117) = 12.85, *p* < .001). Participants in the Healthy Norm condition (*M* = 5.23, *SD* = .64, *t*(78) = 2.22, *p* = .029, *d* = 0.50) and No Norm condition (*M* = 5.24, *SD* = .75, *t*(78) = -2.21, *p* = .03, *d* = -0.50) stated a greater preference for healthy foods than participants in the Unhealthy Norm condition (*M* = 4.83, *SD* = .93). There were no statistically significant differences between the Healthy Norm condition and the No Norm condition for preferences for healthy foods (*p* = .91). Participants in the Unhealthy Norm condition (*M* = 4.91, *SD* = .78, *t*(78) = -4.87, p < .001, *d* = -1.10) and No Norm condition (*M* = 4.68, *SD* = .94, *t*(78) = -3.50, *p* = .001, *d* = -0.79) stated a greater preference for unhealthy foods than participants in the Healthy Norm condition (*M* = 3.91, *SD* = 1.04). There were no statistically significant differences between the Unhealthy Norm condition and the No Norm condition for preferences for unhealthy foods in the first rating session (*p* = .28; Fig 3).

#### Follow-Up Ratings

After establishing that participants shifted their food preferences to align with their norm condition during the norm manipulation, we investigated whether these preference differences continued in the absence of peer feedback. To do this, we first examined the overall pattern of preferences for healthy and unhealthy foods during the follow-up rating session. This session took place 10 minutes after the norm manipulation. Participants rated their preference for a series of healthy and unhealthy images without viewing any peer ratings.

**Fig 4:**
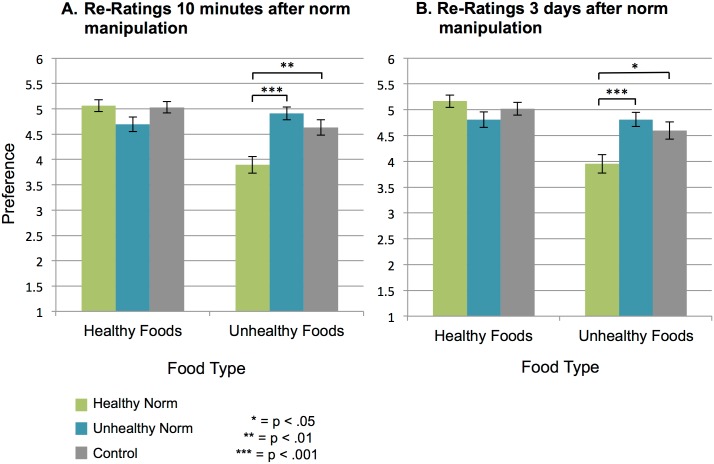
Between-condition preference differences for unhealthy foods persist three days after norm manipulation. Participants in the Healthy Norm condition prefer unhealthy foods less than participants in the Unhealthy Norm condition and No Norm condition. This preference difference persists even three days after the norm manipulation. These graphs show preferences for all food stimuli presented during each rating session (repeated and novel images are collapsed). Error bars represent *SEM*.

There was a statistically significant difference between conditions for preferences for unhealthy foods (*F*(2,117) = 12.38, *p* < .001) but not for healthy foods. Participants in the Healthy Norm condition (*M* = 3.90, *SD* = 1.05) preferred unhealthy foods less than participants in the Unhealthy Norm condition (*M* = 4.91, *SD* = .80, *t*(78) = -4.87, *p* < .001, *d* = -1.10) and No Norm condition (*M* = 4.63, *SD* = .96, *t*(78) = -3.28, *p* = .002, *d* = -0.74). There were no statistically significant differences between the Unhealthy Norm condition and the No Norm condition for preferences for unhealthy foods in the re-rating session (*p* = .19; Fig 4A).

#### Repeated vs. Novel Images

Conformity—participants’ shift in rating to match group preferences for specific food items—likely contributed to differences in food preferences across our healthy and unhealthy norm conditions. However, if people internalized not only item-level information about group preferences, but also *rules* governing group norms, then our participants should exhibit influence even over novel food items that were not initially paired with any peer ratings. To examine this possibility, we investigated whether participants exhibited similar preference differences between conditions when rating novel food items. Although food images for participants in the No Norm condition were never paired with peer feedback, we were still able to compute preferences for repeated images (images that appeared in both the initial and re-rating image sets) and novel images (images that only appeared during the re-rating sets).

Consistent with previous analyses, participants in the Healthy Norm condition (*M* = 3.88, *SD* = 1.11) preferred novel unhealthy foods less than participants in the Unhealthy Norm condition (*M* = 4.93, *SD* = .85, *t*(78) = -4.78, *p* < .001, *d* = -1.08) and No Norm condition (*M* = 4.60, *SD* = 1.03, *t*(78) = -3.04, *p* = .003, *d* = -0.69). There were no statistically significant differences between the Healthy Norm condition and the Unhealthy Norm condition (*p* = .08) or No Norm condition (*p* = .97) for preferences for novel healthy foods.

**Fig 5:**
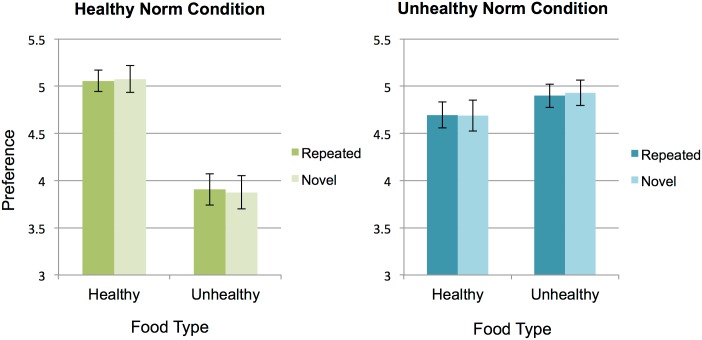
Norm-related preferences spread to novel food images. Within each norm condition, preferences for novel images do not differ from preferences for repeated images. Error bars represent *SEM*.

If participants changed their preferences for novel food images to the same extent that they changed their preferences for repeated food images, there should be no differences in preferences for healthy repeated and novel images and no differences in preferences for unhealthy repeated and novel images within each condition. To test this, we ran a paired-sample t-test between repeated and novel images for each category of images (healthy and unhealthy) for each norm condition. For both the Healthy Norm condition and the Unhealthy Norm condition, there were no differences in ratings between repeated healthy (*M*_*Healthy Norm*_ = 5.06, *SD*_*Healthy Norm*_ = .72; *M*_*Unhealthy Norm*_ = 4.69, *SD*_*Unhealthy Norm*_ = .86) and repeated unhealthy (*M*_*Healthy Norm*_ = 3.91, *SD*_*Healthy Norm*_ = 1.04; *M*_*Unhealthy Norm*_ = 4.90, *SD*_*Unhealthy Norm*_ = .79) images and compared to novel healthy (*M*_*Healthy Norm*_ = 5.08, *SD*_*Healthy Norm*_ = .90, *t*(39) = -.31, *p* = .76, *d* = -0.05; *M*_*Unhealthy Norm*_ = 4.69, *SD*_*Unhealthy Norm*_ = 1.04, *t*(39) = .10, *p* = .92, *d* = .17) and novel unhealthy (*M*_*Healthy Norm*_ = 3.88, *SD*_*Healthy Norm*_ = 1.11, *t*(39) = .45, *p* = .65, *d* = .07; *M*_*Unhealthy Norm*_ = 4.93, *SD*_*Unhealthy Norm*_ = .85, *t*(39) = -.73, *p* = .47, *d* = -0.12; Fig 5) images. The similar pattern of preferences between repeated and novel images indicates that participants extrapolated peer feedback beyond specific items, and instead internalized a social norm *rule* about categories of foods.

#### Persistence of Influence After a 3-day Delay

Next we examined whether the preference differences in the first re-rating session persisted through to the second re-rating session that took place three days after the norm manipulation. We first looked at the overall pattern of preferences for all healthy and unhealthy foods in the final preference-rating session. As with the two previous preference-rating sessions, there was a statistically significant difference between conditions for preferences for unhealthy foods (*F*(2,115) = 7.50, *p* = .001) three days later. Participants in the Healthy Norm condition (*M* = 3.95, *SD* = 1.12) rated unhealthy foods lower than participants in the Unhealthy Norm condition (*M* = 4.81, *SD* = .88, *t*(76) = -3.78, *p* < .001, *d* = -0.87) and No Norm condition (*M* = 4.60, *SD* = 1.05, *t*(77) = -2.64, *p* = .01, *d* = -0.60). There were no statistically significant differences between the Unhealthy Norm condition and the No Norm condition for preferences for healthy foods (*p* = .35; Fig 4B).

Because this final re-rating task included novel and repeated food images, we ran a paired-sample t-test between healthy / unhealthy novel and repeated food images to investigate whether between-condition preference differences held for *all* images, or only those images that were initially paired with peer ratings. For both the Healthy Norm condition and the Unhealthy Norm condition, there were no differences in ratings between repeated healthy (*M*_*Healthy Norm*_ = 5.19, *SD*_*Healthy Norm*_ = .73; *M*_*Unhealthy Norm*_ = 4.83, *SD*_*Unhealthy Norm*_ = .93) and repeated unhealthy (*M*_*Healthy Norm*_ = 3.96, *SD*_*Healthy Norm*_ = 1.12; *M*_*Unhealthy Norm*_ = 4.81, *SD*_*Unhealthy Norm*_ = .88) images and compared to novel healthy (*M*_*Healthy Norm*_ = 5.09, *SD*_*Healthy Norm*_ = .86, *t*(38) = 1.99, *p* = .054, *d* = .32; *M*_*Unhealthy Norm*_ = 4.73, *SD*_*Unhealthy Norm*_ = 1.05, *t*(38) = 1.97, *p* = .06, *d* = .32) and novel unhealthy (*M*_*Healthy Norm*_ = 3.92, *SD*_*Healthy Norm*_ = 1.15, *t*(38) = .72, *p* = .47, *d* = .11; *M*_*Unhealthy Norm*_ = 4.82, *SD*_*Unhealthy Norm*_ = .91, *t*(38) = -.21, *p* = .84, *d* = -0.03) images. This again suggests that participants shifted their food preferences in response to rule based social norms, even three days after the initial norm manipulation.

### Health Perception

We also examined whether there were any differences in health perception between conditions. For each participant, we calculated the average health rating they gave healthy foods and the average health rating they gave unhealthy foods. We then ran a one-way ANOVA to look for differences in health ratings for healthy and unhealthy foods. There was a statistically significant difference between conditions for health ratings of unhealthy foods (*F*(2,114) = 3.62, *p* = .03). Participants in the Unhealthy Norm condition (*M* = 2.40, *SD* = .50) rated unhealthy foods as being significantly healthier than participants in the Healthy Norm condition (*M* = 2.11, *SD* = .56, *t*(75) = -2.42, *p* = .02, *d* = -0.56) and No Norm condition (*M* = 2.11, *SD* = .57, *t*(76) = 2.36, *p* = .02, *d* = 0.54). There were no statistically significant differences between the Healthy Norm condition and the No Norm condition (*p* = .96).

### In-Lab Eating Behavior

Finally, we investigated whether social norms influenced the amount of M&Ms that participants took immediately following the norm manipulation. We used a one-way ANOVA to look for differences in the amount of M&Ms (measured in grams) that participants took in each condition. There were no differences in the amount of M&Ms that participants took (*F*(2,117) = .58, *p* = .56). Participants in the Healthy Norm condition (*M* = 4.90, *SD* = 7.54) did not take significantly less M&Ms than participants in the Unhealthy Norm condition (*M* = 5.80, *SD* = 7.58, *t*(78) = -0.53, *p* = .60, *d* = -0.12) and No Norm condition (*M* = 4.18, *SD* = 4.82, *t*(78) = .51, *p* = .61, *d* = 0.12). There were no statistically significant differences between the Unhealthy Norm condition and the No Norm condition for preferences for healthy foods in the re-rating session (*p* = .27).

## Discussion

The present study adds to our understanding of how social norms impact food preferences in several important ways. Using a novel version of an existing conformity paradigm, we demonstrated that people learn not only to adopt peers’ preferences for *particular* food items. Instead, they appear to internalize a broader ‘health rule’ based on peer ratings, and then apply this rule when judging novel stimuli. This is both theoretically and practically interesting. First, this finding provides insight into how people update their preferences in response to social norm information. In our prior work, participants received social norm information about individual food items, irrespective of a norm rule [22]. There, participants updated their preferences on an item-by-item basis. Our present work demonstrates that when social norm information has an underlying norm rule, participants can update their preferences more globally. Future work should investigate whether participants are similarly able to learn and generalize norm rules about other types of stimuli. Second, this insight suggests that it may be possible to systematically manipulate perceptions of social norms. Rather than communicating a specific norm message (e.g., to conserve electricity or to vote in a particular election) it may be possible to convey more general messages (e.g., to be environmentally friendly or to participate in democracy).

The present study also highlights an interesting asymmetry in how health norms are processed. Participants in the Healthy Norm condition exhibited a decreased preference for unhealthy foods. However, participants in the Unhealthy Norm condition did not exhibit a corresponding decrease in their preferences for healthy foods. Although our study purposefully focused on the role of descriptive social norms, this asymmetry might be explained when considering the role of injunctive social norms [38]. Given that food-based injunctive norms are often health-positive (i.e., people should prefer healthy foods) it is possible that an injunctive norm was implicitly present across all conditions.

Consistent with this prediction, a recent study examined the interplay of injunctive and descriptive norms on intentions to engage in energy conservation [39]. Participants were exposed to one injunctive and one descriptive norm. These norms were either congruent (e.g., both supportive of energy conservation) or conflicted (e.g., one norm was supportive of energy conservation and the other norm was unsupportive). Participants exposed to congruent, supportive norms reported higher intentions to conserve energy than participants exposed to conflicted norms. Similarly, in the present study, participants in the Healthy Norm condition received descriptive information congruent with an implicit injunctive norm. However, participants in the Unhealthy Norm condition received descriptive norm information that conflicted with an implicit injunctive norm. The conflicted norm information in the Unhealthy Norm condition may have weakened the effects of our descriptive social norm manipulation.

The asymmetry in our results might also be a result of using a Stanford population. California is a relatively healthy state [40–41] and Stanford is a particularly healthy university [42]. Though the Stanford student body is diverse, while students are on campus they have easy access to healthy foods. Many studies link the availability of healthful foods to health factors such as diet and obesity [43–45]. Indeed, participants stated a consistently high preference for healthy foods across all conditions. Given that our participants had a strong, pre-existing preference for healthy foods, they may have been unwilling to change this preference, even in response to competing descriptive norm information. Many classic studies on confirmation bias find that people have a tendency to accept evidence that confirms their pre-existing opinions and to be critical of information that goes against these opinions [46]. When people have strong biases (e.g., healthy foods are preferable), social influence in the opposite direction might be less influential. It is possible that we would have seen a greater effect of our norm manipulation—and less asymmetry—in a population that did not have a strong preference for healthy foods. Future studies should investigate how important demographic information—SES, BMI, gender, etc—interacts with this health-based social norm intervention.

The preference differences elicited by our norm manipulation persisted three days later. A recent study using a similar conformity paradigm to investigate changes in ratings of facial attractiveness also found that effects of their social norm manipulation lasted for three days [20], suggesting that persistence is possible in different domains. Again, an important application of this work is whether or not social norms—and this paradigm in particular—might function as an intervention strategy to promote healthy eating. Evidence of persistence in this paradigm is encouraging. A cornerstone of a successful intervention is the ability to improve outcomes long into the future [47].

Our results also highlight a potential limitation this paradigm might have as an intervention strategy. Though participants in the Healthy Norm condition decreased their preferences for unhealthy foods, these participants did not take fewer M&Ms than participants in the other two conditions, suggesting that this norm manipulation might not influence subsequent eating behavior. One explanation could be that participants are only changing their public preferences, not changing the way they privately think about food. People may change their stated preferences simply to avoid social rejection while privately continuing to hold their own preferences [48]. It might also be the case that participants’ eating behavior *did change* as a result of our norm manipulation, but that our measure of eating behavior simply did not capture these changes. Participants may have changed their consumption of healthy foods, experienced changes in their eating behavior several days after the norm manipulation, or adjusted their eating behavior in a myriad of other ways. Future studies should more comprehensively investigate the relation between shifting food preferences and eating behavior. For example, allowing participants to choose from a range of healthy and unhealthy food options would allow us to more precisely determine both the type and quantity of food that participants choose to eat after a social norm manipulation. Of course, even if we conclusively determined that this particular social norm manipulation *never* affects eating behavior this would be an informative null finding. It might be the case that social norm manipulations have to be specific to a particular eating behavior measure (e.g., quantity-based or food choice) in order to effectively alter eating behavior.

The fact that we did observe changes in health *perception* does suggest that participants are indeed internalizing the norm information to some extent. Participants in the Unhealthy Norm condition perceived unhealthy foods as being significantly healthier than the other norm conditions. Because participants never learned social norm information about how others perceived the healthfulness of these food items, it is unclear why we found this difference in health perception and further unclear what consequences this difference could have on participants’ eating behavior. Though we did not find a change in eating behavior directly, many studies underscore the importance of having accurate health perceptions. People who demonstrate more accurate health perception tend to have better nutritional knowledge [49], better diets [27–29], and lower BMI [26]. A change in health perception could lay the foundation for long-term changes in diet. More research will have to be done to investigate the extent to which these particular changes in health perception influence later eating behavior.

Finally, it is important to note that this social norm manipulation might have influenced eating behavior in a population *motivated* to change their eating behavior. Importantly, our sample was restricted to non-dieters, a group that presumably has little motivation to change their eating behavior. Dieters differ from non-dieters in important ways. For example, dieters have been shown to categorize food in terms of ‘guilt’ more readily than non-dieters [50]. For a population of dieters, our manipulation might have been even more salient and more likely to lead to changes in eating behavior. Using health-based norm manipulations might be a fruitful way to explore many complexities of social norm transmission.

Social norms can powerfully change people’s food preferences but their effects on subsequent eating behavior may be more limited. Given the social nature of eating, it is likely that social norms do influence eating behavior in some capacity and future research should continue to explore this relationship.
